# Is IVF/ICSI an Independent Risk Factor for Spontaneous Preterm Birth in Singletons? A Population-Based Cohort Study

**DOI:** 10.1155/2018/7124362

**Published:** 2018-12-30

**Authors:** Nina Jančar, Barbara Mihevc Ponikvar, Sonja Tomšič, Eda Vrtačnik Bokal, Sara Korošec

**Affiliations:** ^1^Department of Human Reproduction, Division of Obstetrics and Gynaecology, University Medical Centre Ljubljana, Slovenia; ^2^Health Survey and Health Promotion Department, National Institute of Public Health, Ljubljana, Slovenia

## Abstract

The aim of our study was to explore the risk factors for very preterm (gestation under 32 weeks) and moderate preterm birth (gestation weeks 32-36 6/7) in singleton pregnancies in a national retrospective cohort study. We also wanted to establish whether IVF/ICSI is an independent risk factor for preterm birth after adjusting for already known confounders. We used data for 267 718 singleton births from 2002-2015 from the National Perinatal Information System of Slovenia, containing data on woman, pregnancy, birth, the postpartum period, and the neonate for each mother–infant pair. Mode of conception, maternal age, education, BMI, parity, smoking, history of cervical excision procedure, history of hysteroscopic resection of uterine septum, presence of other congenital uterine malformations, bleeding in pregnancy, preeclampsia or HELLP and maternal heart, and pulmonary or renal illness were included in the analyses. Unadjusted OR for very preterm birth after IVF-ICSI was 2.8 and for moderate preterm birth was 1.7. After adjusting for known confounders, the OR was still significantly elevated (1.6 and 1.3, respectively). Risk factors for very preterm birth with OR higher than 2.4 were history of cervical excision procedure, resection of uterine septum, operation or having other congenital uterine malformations, and bleeding in pregnancy. Risk factors for very preterm birth with OR between 1.4 and 2.1 were age >35 years, being underweight or obese, not having professional education, smoking, first birth, preeclampsia/HELLP, and IVF/ICSI. Risk factors for moderate preterm birth with OR higher than 2.4 were history of cold knife conization and other congenital uterine malformations. We found that even after adjustment, IVF/ICSI represents a single risk factor for early and late preterm birth even after adjustment with other risks such as maternal age, smoking, or a history of invasive procedures for either cervical intraepithelial neoplasia or infertility treatment.

## 1. Introduction

Assisted reproductive technology is now widely used for treatment of different female and male causes of infertility. More and more babies are born after IVF/ICSI procedures each year worldwide. The same is true for Slovenia, where up to 4% of babies are born after IVF yearly [1]. IVF was first found to be connected with preterm birth, predominately because of increased percentage of multiple pregnancies. With the preferred use of single embryo transfer, the percentage of multiple gestations was significantly reduced [2]. But even singleton pregnancies after IVF were found to be connected with preterm birth [3–9].

Multiple factors were found so far to be connected with preterm birth, the most prevalent being extremes of maternal age, low maternal BMI, maternal smoking, infections, history of cervical excision procedures, uterine anomalies, infertility treatment, and others [10, 11].

Women conceiving after IVF/ICSI are a special population of pregnant women. Due to many years of infertility, they are older than women conceiving spontaneously. The infertility itself is a known risk factor for preterm birth [12], since different disorders (endometriosis, adenomyosis, polycystic ovary syndrome, and uterine fibroids) and unexplained infertility share inflammatory pathways, hormonal aberrations, decidual senescence and vascular abnormalities that may impair pregnancy success through common mechanisms [13]. These patients also have a history of several gynecologic operations before their pregnancy.

The aim of our study was to explore the risk factors for very preterm birth, before 32 weeks of gestation, and moderate preterm birth, from 32 to 36 6/7 weeks of gestation, in singleton pregnancies in a large 14-year national study. We wanted also to further explore weather pregnancy after IVF/ICSI is an independent risk factor for very preterm birth and moderate preterm birth after adjusting for already known important confounders.

## 2. Materials and Methods

We conducted a population-based retrospective cohort study using data from Medical Birth Registry, the National Perinatal Information System of Slovenia (NPIS). We followed the methods of Jančar et al. 2016 [14]. NPIS contains data on woman, pregnancy, birth, the postpartum period, and the neonate for each mother–infant/infants pair. Data is collected at the time of birth in all 14 maternal hospitals in Slovenia according to standardized methodology and premade definitions of over 100 different social, health, and perinatal variables [15]. The National Perinatal Information System of Slovenia includes all live deliveries regardless of child's gestation and birth eight. Besides, all stillborn with birthweight of at least 500 g or gestational age of at least 22 weeks or both are included in the system. Registration is mandatory by law since NPIS also serves as Slovenia's medical birth registry. Data is sent to the Slovenian National Institute of Public Health on a yearly basis, where it goes through statistical quality checks, is edited, and forms the basis for the official perinatal statistics of Slovenia. In the study period 99.9% of women were delivered in a hospital.

This retrospective cohort did not need ethical approval according to Slovenian law [16].

The study population consisted of Slovenian residents who gave birth to singletons from January 1, 2002, to December 31, 2015. In this period there were 282 517 births in Slovenia. After exclusion of foreigners, Slovenian residents had 281 358 births in this period. After exclusion of 9800 multiple gestations (3.5% of all births), 3 836 (1.4%) induced births and elective cesarean sections before 37 weeks of gestation due to maternal and fetal conditions, and 4 cases with gestational week at the time of birth not recorded, our final sample for the analysis consisted of 267 718 spontaneous births of singletons.

To be able to analyze factors associated with spontaneous preterm birth, we excluded all induced births and elective cesarean sections before 37 weeks of gestation, which were carried out due to maternal or fetal illness or condition, such as preeclampsia, maternal chronical illness, intrauterine growth restriction, or other critical conditions. Only births with a spontaneous onset have been included.

Outcome variables were spontaneous preterm birth before 32 weeks and spontaneous preterm birth between 32 and 36 6/7 weeks of gestation. Gestational age was determined according to the last menstrual cycle and first ultrasound in pregnancy by obstetrician or gynecologist, in agreement with the paediatrician assessment after birth. In cases of unclear gestational age, it was determined individually, considering the anamnesis of last menstrual period, including possible alterations such as irregular cycles, combined with the first available ultrasound estimation and findings of the pediatrician. The most probable gestational age was included in the system.

Along with mode of conception, IVF/ICSI or spontaneous, a total of 11 other covariates obtained from NPIS were included in the analyses: maternal age, maternal education, maternal BMI, parity, smoking during pregnancy, history of cervical excision procedure, history of hysteroscopic resection of uterine septum, presence of other congenital uterine malformations, bleeding in pregnancy, preeclampsia or HELLP in pregnancy and maternal heart, and pulmonary or renal illness. We selected those covariates, because they have been previously reported to affect the risk for preterm birth [10]. Maternal age was categorized into five groups: younger than 25 years, 25–29 years, 30–34 years, 35–39 years, and 40 years or older. Maternal education was categorized into five groups: primary or less, vocational, secondary or professional, tertiary, and not stated. Maternal BMI was categorized into four groups: less than 18.5 kg/m^2^, 18.5–24.9 kg/m^2^, 25–29.9 kg/m^2^, and 30 kg/m^2^ and higher. Parity was categorized into three groups: first birth, second birth, and third or more. Reported smoking during pregnancy was categorized into two groups: no or yes. History of cervical excision procedure was categorized into three groups: no, history of cold-knife conization, history of newer excision procedures, and predominately large loop excision of transformation zone (LLETZ). History of hysteroscopic resection of uterine septum was categorized into two groups: no or yes. Presence of other congenital uterine malformation was categorized into two groups: no, when there was no anomaly, and yes, when it was present or when it had been surgically corrected before this pregnancy. Bleeding in pregnancy was categorized into two groups: no and yes, when there had been a history of bleeding anytime in this pregnancy. Preeclampsia or HELLP in this pregnancy was categorized into two groups: no or yes. A history of maternal heart and pulmonary or renal illness was categorized into two groups: no or yes.

Chi-square test was used for descriptive analysis. Logistic regression analyses were performed to estimate crude odds ratio (OR) and adjusted odds ratio (aOR) and their 95% confidence intervals (95% CI) with two-sided probability (*p*) values. A* p* value of < 0.05 was considered statistically significant. For statistical calculations, we used IBM SPSS Statistics for Windows, Version 21.0. (Armonk, NY: IBM Corp.).

## 3. Results

In the study period a total of 5 837 (2.2%) singletons were conceived after IVF/ICSI. The percentage of pregnancies after IVF/ICSI rose constantly over the years: from 1.5% in the year 2002 to 3.9% in the year 2015. The characteristics of our study population according to distribution of covariates divided by mode of conception is shown in Table 1.

A total of 11 605 singleton births (4.3%) in our population were premature, before 37 weeks of gestation. The distributions of preterm births according to gestation and mode of conception are also shown in Table 1.

We have calculated the unadjusted odds ratios (OR) for very preterm birth, before 32 weeks of gestation and for moderate preterm birth, between 32 and 36 6/7 of gestation, for women, who conceived after IVF-ICSI, compared to women conceiving spontaneously. Unadjusted OR for very preterm birth in pregnancies after IVF-ICSI conception was 2.8, for moderately preterm birth 1.7. After adjusting for included known confounders, the OR remained statistically significantly elevated. The results are presented in Table 2.

We also prepared multivariate analysis of different factors contributing to premature birth in our study population taking into account twelve covariates: mode of conception, maternal age, maternal education, maternal BMI, parity, smoking during pregnancy, history of cervical excision procedure, history of hysteroscopic resection of uterine septum, presence or previous surgical correction of other congenital uterine malformation, bleeding in pregnancy, preeclampsia or HELLP in pregnancy and a history of maternal heart, and renal or pulmonary illness. The risk factors for very preterm birth, before 32 weeks, and moderate preterm birth, between 32 and 36 6/7, are presented in Table 3.

According to this multivariate analysis, there are some factors contributing to the risk for very preterm birth in our population. Those factors are age more than 35 years, being underweight or obese, not having any professional education, smoking during pregnancy, first birth, bleeding, preeclampsia or HELLP during this pregnancy, and pregnancy after IVF/ICSI, which all have OR between 1.4 and 2.1. Similarly, factors for moderate preterm birth between 32 and 36 6/7 having OR between 1.4 and 2.1 are age more than 40 years, being underweight, first birth, LLETZ, and bleeding in pregnancy.

The most important risk factors for very preterm birth, which have OR higher than 2.4, are having a history of any cervical excision procedure, having previous hysteroscopic resection of uterine septum, having or being previously operated for other congenital uterine malformation, or bleeding any time in pregnancy. The most important risk factors for moderate preterm birth which have OR higher than 2.4, are having a history of any cervical excision procedure, having previous hysteroscopic resection of uterine septum, and having or being previously operated for other congenital uterine malformation.

## 4. Discussion

### 4.1. Principal Findings

Our results showed that the risk of sPTB in singleton pregnancies after IVF/ICSI is significantly greater than that in spontaneously conceived singletons. Our findings are in agreement with the most recent meta-analysis of Cavoretto et al. [17]. In this meta-analysis it has been stated that the findings should be interpreted with caution given the low quality of the available evidence. Therefore, our results could be of importance for clinicians to increase their surveillance in these patients.

As presented in Table 1, there are some important differences between women giving birth after IVF/ICSI conception and spontaneous conception. In the IVF/ICSI group the proportion of women under 30 years was three times lower (18.6% versus 52.5%; p < 0.001). Similarly, there were substantially more women aged 35 and more in the IVF/ICSI group (38.6% versus 14.1% p < 0.001).

For women, who conceive after IVF/ICSI, it is significantly more likely that they are nulliparous and have significantly more cervical excision procedures, significantly more resections of uterine septum, and significantly more other congenital malformations than women who conceive spontaneously. Furthermore, they are more likely to bleed at any time in pregnancy and to have other complications such as preeclampsia and HELLP than women, who conceive spontaneously. Therefore we performed an adjusted OR analysis. In this analysis, the OR for very preterm and moderate preterm birth remained significantly higher, which implies that IVF/ICSI might be an independent risk factor for preterm birth. Underweight patients in our analysis have higher OR for preterm birth than overweight or obese patients, which has been described before [18]. Regarding other included covariates in the analysis we have found that history of cervical excision procedure and history of uterine anomaly or hysteroscopic resection of uterine septum had an important increased OR for preterm birth.

Cold knife conisation and LLETZ were already shown to be important independent risk factors for premature birth in our recent population-based cohort study [14] and in some other studies [19–21]. Previous studies [3–9, 12] have shown that IVF treatment also increases the risk for preterm birth. If a woman has a history of surgical treatment for CIN combined with IVF, the risk for preterm birth was shown to be three times higher in study by Jakobsson et al. [11]. Similar results were obtained also with our present population-based study. Nevertheless, if a woman has high-grade squamous intraepithelial lesion, it has to be treated regardless of pregnancy planning, in order to prevent the development of cervical cancer, whereas low-grade squamous intraepithelial lesions can be left untreated and followed up by colposcopy. It has been shown that the risk for spontaneous abortion is decreased, if pregnancy occurs more than 12 months after the treatment procedure for cervical precancerous lesion, whereas the risk for preterm birth was not affected by this time interval [22, 23].

Hysteroscopic resection of uterine septum was performed in 18.6% women (n = 1084) after IVF/ICSI and in only 3.5% of women (n = 9 111) conceiving spontaneously (p < 0.001). This is the consequence of diagnostic and operative interventions to find the exact cause of infertility before sending the infertile couple to IVF/ICSI. In Slovenia, we tend to have a holistic approach to infertility and we try to treat it in causative manner. Therefore, if we do not find a clear cause of infertility, such as severe teratozoospermia or blocked fallopian tubes, we perform diagnostic hysteroscopy and laparoscopy [24]. If a uterine septum is found during hysteroscopy, we perform a resection at the same time. Furthermore, we perform a diagnostic hysteroscopy also after several embryo transfers of top quality embryos without pregnancy.

Those are the reasons for such a big discrepancy between the two groups of women. It is known that the presence of untreated uterine anomalies is connected to preterm birth [25]. The hysteroscopic surgery of subseptate or septate uterus itself may not restore all aspects of uterine performance, but, according to the literature, it helps to reduce the risk of pregnancy loss and preterm birth [25–28], whether the hysteroscopy plays solely circumstantial or sometimes causative role in preterm birth remains unclear. Since the cervical canal has to be dilated at hysteroscopy, smaller diameter instruments in combination with prostaglandin cervical priming are being used currently [29].

Due to these findings, the procedures on cervix and uterus should be clearly indicated, especially in infertile population, since they might change integrity of the cervix in subsequent pregnancy.

Bleeding any time in pregnancy was also more common in the group of women after IVF/ICSI. It is already known that bleeding and spontaneous abortion in first trimester occur more common after IVF/ICSI than after spontaneous conception. To some extent, this can be contributed to higher aneuploidy rate due to the advanced age of both partners, mostly female, but also male partner [30, 31]. On the other hand, the surplus transferred and implanted embryos could be degraded, which is known as “the vanishing twin syndrome”. There is substantial evidence that very preterm birth is connected to vanishing of one gestational sac [32].

Placenta previa and certain other placental abnormalities, followed by antepartum hemorrhage, generally occur more often in pregnancies after IVF and the causes are still obscure [33–36]. Other placentation abnormalities, probably connected with pregnancies after IVF, are placenta accreta [37, 38], vasa previa [39, 40], and abnormal umbilical cord insertion [41]. All these facts have been clearly visible also in our study, where bleeding in any trimester of pregnancy was twice more common after IVF/ICSI than in spontaneous pregnancies.

Preeclampsia is also more common in pregnancies after IVF, mostly due to infertile population attributes, since after adjustment for the confounders the association was weak [42].

### 4.2. Meaning of the Findings, Clinical Implications

This study confirms findings that the population of women, who conceive after IVF/ICSI, is different than the population of women, conceiving spontaneously, and they deserve a closer and more dedicated follow-up during their long wanted pregnancy.

Furthermore, in this large cross-sectional study we proposed a combination of risk factors that define population of women with highest risk for spontaneous onset of preterm delivery.

### 4.3. Research Implications

According to the findings, in IVF patients, the cervical neoplasia treatment methods and hysteroscopic operation techniques should have been analyzed further, to narrow groups with the highest preterm birth risk. The cervical ostium assessment prior to hysteroscopy could be of some importance.

Besides, different IVF treatment methods should have been analysed, since the perinatal outcome is not the same in all cases: children, born after fresh ET, are at higher risk for low birthweight and premature delivery as children, born after frozen-thawed ET [43, 44].

In our study no late abortions with stillborn fetuses under 500 g and before 22 gestational weeks were included. This data could possibly show another distribution of risk factors.

There are still numerous known preterm birth risk factors, including previous preterm birth, asymptomatic bacteriuria, sexual intercourse, and psychiatric disorders, which have not been analyzed or added to our model and could add an insight to the etiology of preterm birth.

### 4.4. Strength and Weaknesses

Our study was designed as a population-based cohort study. The main advantage of a population-based study over 14 years is the large number of births available for analysis and inclusion of all population subgroups (by social class, by region, by life-style, by religion, etc.). In Slovenia, there is a universal access to assisted reproductive procedures, regardless of social status. The costs for six cycles of IVF for the first child and four cycles for the second or third child are covered by medical insurance for every infertile couple, where another treatment of infertility was not successful or not possible. This fact and total population inclusion diminishes the existence of selection bias in our study.

We realize that by employing data from administrative sources one could question the quality of such data. As described, the data source is obligatory by law, is in use for more than 30 years in Slovenia, is predefined, has regular quality checks, and is made of data gathered in medical records which are produced by medical staff in maternity hospitals; this is why we consider our data to be of reasonably good quality.

One of the weaknesses of our study that was already mentioned is the fact that no late abortions were included in the analysis.

## 5. Conclusion

In this large cross-sectional national study we proposed a combination of risk factors that define population of women with higher risk for preterm birth. We found that even after adjustment IVF/ICSI represents a single risk factor for early and late spontaneous preterm birth even after adjustment with other risks such as maternal age, smoking, or a history of invasive procedures for either cervical intraepithelial neoplasia or infertility. Therefore, these women deserve a more close and dedicated follow-up during their pregnancy.

## Figures and Tables

**Table 1:** Characteristics of women included in the analysis, Slovenia, 2002–2015.

***Characteristic***	***Spontaneous conception***		***IVF/ICSI***		***p value***	***All births***	
	***n = 261 881***		***n = 5 837***			***n = 267 718***	
***Maternal age (years)***	***n***	%	***N***	%	***<0.001***	***n***	%
< 25	40150	15.3	79	1.4		40 229	15.0
25 – 29	97348	37.2	1006	17.2		98 354	36.7
30 – 34	87586	33.4	2496	42.8		90 082	33.6
35 – 39	31625	12.1	1782	30.5		33 407	12.5
40 ≥	5172	2.0	474	8.1		5 646	2.1
***Maternal education***					***<0.001***		
Primary or less	11 174	4,3	197	3.4		11 371	4.2
Vocational	35 525	13,6	779	13.3		36 304	13.6
Secondary or professional	89 836	34,3	1 849	31.7		91 685	34.2
Tertiary	92 322	35,3	2 599	44.5		94 921	35.5
Not stated	33 024	12.6	413	7.1		33 437	12.5
***Maternal BMI***					***<0.001***		
< 18.5 kg/m^2^	13513	5.2	233	4.0		13 746	5.1
18.5–24.9 kg/m^2^	180295	68.8	3891	66.7		184 186	68.8
25–29.9 kg/m^2^	46616	17.8	1137	19.5		47 753	17.8
30 kg/m^2^≥	21362	8.2	574	9.8		21 936	8.2
Missing data	95	0.0	2	0.0		97	0,0
***Parity***					***<0.001***		
0	127684	48.8	4345	74.4		132 029	49.3
1	97868	37.4	1353	23.2		99 221	37.1
2≥	36329	13.9	139	2.4		36 468	13.6
***Smoking during pregnancy***					***<0.001***		
No	233049	89.0	5368	92.0		238 417	89.1
Yes	28832	11.0	469	8.0		29 301	10.9
***Cervical excision procedure***					***<0.001***		
No	255164	97.4	5566	95.4		260 730	97.4
Cold-knife	2570	1.0	119	2.0		2 689	1.0
Other – LLETZ	4147	1.6	152	2.6		4 299	1.6
***Resection of uterine septum***					***<0.001***		
No	252770	96.5	4753	81.4		257 523	96.2
Yes	9111	3.5	1084	18.6		10 195	3.8
***Other uterine malformation***					***<0.001***		
No	260118	99.3	5744	98.4		265 862	99.3
Yes	1763	0.7	93	1.6		1 856	0.7
***Bleeding in pregnancy***					***<0.001***		
No	243991	93.2	5035	86.3		249 026	93.0
Yes	17890	6.8	802	13.7		18 692	7.0
***Preeclampsia / HELLP***					***<0.001***		
No	257528	98.3	5685	97.4		263 213	98.3
Yes	4353	1.7	152	2.6		4 505	1.7
***Maternal heart, renal or pulmonary illness***					*0,181*		
No	257136	98.2	5745	98.4		262 881	98.2
Yes	4745	1.8	92	1.6		4 837	1.8
***Gestational age at birth (weeks)***					***<0.001***		
< 28	761	0.3	56	1.0		817	0.3
28 to 31 and 6/7	950	0.4	47	0.8		997	0.4
32 to 33 and 6/7	1229	0.5	48	0.8		1 277	0.5
34 to 36 and 6/7	8216	3.1	298	5.1		8 514	3.2
37 ≥	250725	95.7	5388	92.3		256 113	95.7

BMI: body mass index.

LLETZ: large loop excision of transformation zone.

HELLP syndrome: syndrome with hemolysis, elevated liver enzymes, and low platelet count.

**Table 2:** Unadjusted and adjusted*∗* odds ratio (OR) for spontaneous preterm birth at different gestations for women conceiving after IVF/ICSI compared to women conceiving spontaneously, Slovenia, 2002–2015.

Gestation (weeks)	Unadjusted odds ratio	Confidence interval	p value	Adjusted*∗* odds ratio	Confidence interval	p value
< 32	2.801	2.292 – 3.424	< 0.001	1,555	1.256 – 1.925	< 0.001
32 to 36 and 6/7	1.705	1.526 – 1.904	< 0.001	1,300	1.159 – 1,459	< 0.001

*∗* Adjusted for 11 covariates: maternal age, maternal education, maternal BMI, parity, smoking during pregnancy, history of cervical excision procedure, history of hysteroscopic resection of uterine septum, presence of other congenital uterine malformations, bleeding in pregnancy, preeclampsia or HELLP in pregnancy and maternal heart, and pulmonary or renal illness.

**Table 3:** Multivariate analysis of risk factors for spontaneous preterm birth before 32 weeks and between 32 and 36 6/7 weeks of gestation, Slovenia, 2002–2015.

Preterm birth	before 32 weeks				between 32 to 36 and 6/7 weeks			
	Odds ratio	95% confidence interval		p value	Odds ratio	95% confidence interval		p value
Covariates		Lower	Upper			Lower	Upper	
Maternal age < 25								
25–29 years	1,025	0,878	1,196	0,758	1,031	0,966	1,099	0,358
30–35 years	1,127	0,956	1,329	0,153	1,106	1,032	1,185	0,005
35–39 years	1,496	1,237	1,809	< 0,001	1,309	1,204	1,424	< 0,001
40 ≥	1,975	1,491	2,617	< 0,001	1,594	1,390	1,828	< 0,001
Maternal education: Tertiary								
Secondary or professional	1,232	1,098	1,382	<0,001	1,074	1,020	1,13	0,006
Vocational	1,282	1,102	1,491	0,001	1,148	1,073	1,228	< 0,001
Primary or less	1,801	1,456	2,227	< 0,001	1,423	1,289	1,572	< 0,001
Not stated	0,712	0,588	0,861	< 0,001	1,077	1,005	1,154	0,037
Second birth								
First birth	1,443	1,293	1,611	<0,001	1,426	1,36	1,496	< 0,001
Third birth or more	1,143	0,979	1,335	0,092	1,093	1,019	1,171	0,012
Smoking during pregnancy	1,445	1,265	1,650	<0,001	1,313	1,237	1,395	< 0,001
BMI 18.5 – 24.9 kg/m^2^								
BMI < 18.5 kg/m^2^	1,604	1,341	1,917	<0,001	1,456	1,346	1,576	< 0,001
BMI 25 – 29.9 kg/m^2^	1,021	0,9	1,158	0,749	0,935	0,884	0,989	0,018
BMI 30 kg/m^2^ ≥	1,207	1,025	1,422	0,024	0,918	0,849	0,994	0,034
Heart, renal, pulmonary illness	1,255	0,928	1,698	0,141	1,135	0,986	1,308	0,078
Pregnant after IVF/ICSI	1,555	1,256	1,925	< 0,001	1,300	1,159	1,459	< 0,001
No cervical excision procedure								
Cold knife conization	6,162	5,024	7,557	< 0,001	2,455	2,137	2,821	< 0,001
Other – LLETZ	2,735	2,162	3,460	< 0,001	1,770	1,56	2,007	< 0,001
Resection of uterine septum	2,858	2,454	3,328	< 0,001	1,369	1,249	1,500	< 0,001
Other uterine malformation	2,401	1,690	3,413	< 0,001	2,404	2,043	2,828	< 0,001
Preeclampsia / HELLP	1,595	1,199	2,122	0,001	1,316	1,144	1,513	< 0,001
Bleeding in pregnancy	3,078	2,734	3,465	<0,001	1,853	1,74	1,974	< 0,001

## Data Availability

The data from Medical Birth Registry, the National Perinatal Information System of Slovenia, was used to support the findings of this study. These administratively collected anonymous entries of data on Slovenian residents have not been made available since they are protected by the law.
