# Corrigendum to “Is IVF/ICSI an Independent Risk Factor for Spontaneous Preterm Birth in Singletons? A Population-Based Cohort Study”

**DOI:** 10.1155/2019/4940314

**Published:** 2019-04-03

**Authors:** Nina Jančar, Barbara Mihevc Ponikvar, Sonja Tomšič, Eda Vrtačnik Bokal, Sara Korošec

**Affiliations:** ^1^Department of Human Reproduction, Division of Obstetrics and Gynaecology, University Medical Centre Ljubljana, Slovenia; ^2^Health Survey and Health Promotion Department, National Institute of Public Health, Ljubljana, Slovenia

In the article titled “Is IVF/ICSI an Independent Risk Factor for Spontaneous Preterm Birth in Singletons? A Population-Based Cohort Study” [1], the acronym “ISCI” has been corrected to “ICSI” in the title and throughout the article. This correction has been applied in place.
